# Development and Internal Validation of Multivariable Prediction Models for Depression, Anxiety, and Stress Among Saudi Adolescent Video Game Players: A Cross-Sectional Study

**DOI:** 10.3390/bs16101731

**Published:** 2026-09-23

**Authors:** Abdullah Asiri, Ali Alshahrani, Hassan Korairi, Mohammed Aleisa, Faris Alzahrani, Omer Al Kuraydis, Saif Garmush, Hussain Alshatwi, Adel AlAdraj, Hasan Hommadi, Noha Hassanien

**Affiliations:** Preventive Medicine Department, Armed Forces Hospital Southern Region, Ministry of Defense Health Services, Khamis Mushait 62413, Saudi Arabia

**Keywords:** depression, anxiety, stress, video game addiction, prediction model

## Abstract

Background: Anxiety, stress, and depression are increasingly prevalent mental health problems among adolescents, and several studies have reported significant associations between these conditions and video game addiction. The present study was therefore conducted to develop and internally validate multivariable prediction models based on gaming-related and demographic factors in order to identify Saudi adolescent video game players with concurrent moderate-to-extremely severe depression, anxiety, and stress symptoms. Methods: A school-based cross-sectional study was conducted among adolescents. A multistage random sampling technique was used to select 466 participants. Data were collected using a structured questionnaire comprising the Arabic version of the Depression Anxiety Stress Scale (DASS-21) and the validated Arabic version of the 21-item Gaming Addiction Scale (GAS). The prevalence of moderate-to-extremely severe depressive symptoms was 29.2%, whereas the prevalence of moderate-to-extremely severe anxiety and stress symptoms was 47.6% and 27.5%, respectively. Multivariable logistic regression analysis was performed to identify the factors associated with the three mental health outcomes and to derive prediction models and concurrent risk scores. Model performance was evaluated in terms of discrimination and calibration, and the developed risk scores were internally validated using bootstrap resampling. Results: Female gender, prolonged gaming duration (>4 h/day), and playing violent video games were significantly associated with all three mental health outcomes, namely moderate-to-extremely severe depression, anxiety, and stress symptoms, whereas additional factors were independently associated with each individual outcome. Overall, the three internally validated models demonstrated acceptable-to-good discrimination and generally satisfactory calibration. Conclusion: The internally validated models show potential as screening tools for identifying adolescent video game players with concurrent moderate-to-extremely severe depression, anxiety, and stress symptoms on the basis of a combination of demographic and gaming-related characteristics. However, external validation and prospective evaluation is required before they can be applied in clinical or public health practice.

## 1. Introduction

Video gaming (VG) has become the predominant leisure activity during adolescence, a critical developmental period marked by substantial physical, emotional, and social change. During this stage, individuals are particularly vulnerable to mental health problems, and increased engagement in video games may exacerbate these vulnerabilities and may even be associated with addictive behavior (Coutelle et al., 2024).

Mental health professionals regard video game addiction as a functional and behavioral disorder. Two alternative terms have been used in the literature to describe this condition: gaming disorder and internet gaming disorder. Gaming disorder is defined by the World Health Organization and listed in the 11th revision of the International Classification of Diseases (ICD-11) as “a pattern of gaming behavior characterized by impaired control over gaming, increasing priority given to gaming over other activities to the extent that gaming takes precedence over other interests and daily activities, and continuation or escalation of gaming despite the occurrence of negative consequences” (ICD-11: International Classification of Diseases and Related Health Problems 11th Revision, WHO, 2019). Similarly, the American Psychiatric Association’s Diagnostic and Statistical Manual of Mental Disorders defines internet gaming disorder (IGD) as “the persistent and recurrent use of the internet to engage in games, often with other players, leading to clinically significant impairment or distress” (APA, 2013; Petry et al., 2015).

The prevalence of video game addiction among adolescents is increasing globally, although estimates vary widely across countries owing to the different assessment tools used in the literature. A systematic review reported a pooled prevalence of 8.9% across 155 studies (Gao et al., 2022). In Saudi Arabia, reported estimates range from 3.5% to 62.1%, and this wide range precludes a clear conclusion as to whether VG addiction constitutes a problem among Saudi adolescents (Abolfotouh & Barnawi, 2024; Alfaleh et al., 2024; Alghamdi & Alghamdi, 2023; Alrahili et al., 2023; Bumozah et al., 2023).

The literature has documented several adverse consequences of VG addiction among adolescents, including poor academic performance (Gu et al., 2024; Sun et al., 2023) and impairment in both physical and mental health (Sun et al., 2023; Szász-Janocha et al., 2023). In addition, addicted gamers experience social isolation, poor relationships with their families, and impaired social skills (Fan & Zhang, 2023); reduced self-care (Kim et al., 2022); and sleep disturbance (Mohd Noor et al., 2021). The rapid increase in the availability of video games has led to more addictive behavior among adolescents, accompanied by a rise in the associated negative consequences, particularly those affecting mental health (European Commission, Joint Research Centre, 2023).

The relationship between gaming addiction and mental health disorders such as stress, anxiety, and depression is complex. A conceptual framework for this relationship is provided by the Cognitive Behavioral Model (CBM), which posits that maladaptive behaviors such as excessive engagement in video gaming lead to addiction, which in turn disrupts adolescents’ sleep and social relationships. These disruptions impair mental health and contribute to stress, anxiety, and depression (Gursesli et al., 2025).

The rise in mental health disorders among adolescents, particularly anxiety, stress, and depression, together with evidence of their significant relationship with gaming addiction, has been documented in several published studies (Anderson et al., 2017; Cerniglia et al., 2017; Hygen et al., 2020; Long et al., 2018; Mihara & Higuchi, 2017; Narita et al., 2025). Recent systematic reviews further reinforce the evidence for this association (Ghali et al., 2023; Limone et al., 2023; Marques et al., 2023). However, most of these studies have focused on the prevalence of VG addiction and its associated factors. Existing prediction models have primarily aimed at identifying individuals at risk of developing VG addiction itself rather than at predicting mental health outcomes among gamers. Others have examined a single mental health outcome or have relied on machine learning for prediction (Brown et al., 2024; Jahanbakhshi et al., 2025). Therefore, there is a need to develop prediction models for anxiety, stress, and depression among adolescent video game players using readily obtainable demographic and gaming-related characteristics. Such models could translate established risk-factor associations into practical individualized risk estimates and support targeted mental health screening. This gap is particularly relevant in Saudi Arabia, where adolescent gaming is common but locally developed and internally validated models for identifying depression, anxiety, and stress among adolescent video game players remain scarce. Accordingly, the present study aimed to develop and internally validate multivariable models for identifying adolescent video game players with a higher probability of concurrent moderate-to-extremely severe depression, anxiety, and stress symptoms. Such an approach translates research findings into actionable and targeted preventive strategies.

**Hypothesis** **1** **(H1).** *A combination of demographic and gaming-related characteristics can significantly discriminate between adolescent video game players with and without a moderate-to-extremely severe burden of depression, anxiety, and stress symptoms.*

## 2. Materials and Methods

### 2.1. Study Design and Setting

A school-based cross-sectional study was conducted among adolescents attending intermediate and secondary schools in the Aseer region, Saudi Arabia.

### 2.2. Study Population and Sampling

Adolescents aged 12 to 18 years were selected using a multistage random sampling technique. First, the Aseer region was selected from the three administrative provinces of the southern region of the Kingdom of Saudi Arabia through simple random sampling (SRS). Second, a list of all intermediate and secondary schools for both boys and girls in the region was obtained from the educational authorities. From this sampling frame, 12 schools were selected through SRS, comprising six boys’ schools and six girls’ schools. Within each selected school, one class was randomly selected from each grade level, yielding 36 classes. Eligible students in the selected classes were screened for video-gaming status, and those who reported playing video games were invited to participate. A total of 500 eligible adolescent video game players were invited, of whom 466 eligible adolescent video game players completed the questionnaire and were included in the final analysis, giving a response rate of 93.2%. The inclusion criteria were Saudi intermediate and secondary school students who reported playing video games and provided complete responses to the study questionnaire. The exclusion criteria were students who did not report playing video games, had a previously diagnosed psychiatric disorder, or submitted incomplete questionnaire responses.

The potential impact of clustering was assessed using the intraclass correlation coefficient (ICC). The class-level ICCs were very small for stress (ICC = 0.001), anxiety (ICC = 0.002), and depression (ICC = 0.003), and the corresponding design effects were 1.016, 1.029, and 1.033, respectively. These values are very close to 1, indicating minimal variance inflation attributable to class-level clustering. Clustering was therefore considered negligible and had no appreciable effect on the standard errors or on the conclusions of the study.

The minimum required sample size was calculated as 384 students using Epi Info 7 software, assuming a prevalence of mental health problems of 50% and a precision of 5%. The sample was increased to 500 to account for potential non-response. This sample size is adequate for prediction model development, ensuring a sufficient number of events per variable (EPV ≥ 10) for the approximately 11 candidate predictors considered, thereby supporting stable and reliable parameter estimates (Riley et al., 2020).

### 2.3. Study Variables and Data Collection Procedure

Data were collected between December 2023 and February 2024 using structured, self-administered questionnaires completed in the classroom. Prior to data collection, a pilot study was conducted with 50 students to assess the clarity, feasibility, and completion time of the questionnaire; feedback was obtained from the participants, and minor modifications of wording were made accordingly. The pilot data were not included in the analysis.

Outcome Measures: The primary outcomes were symptoms of depression, anxiety, and stress, assessed using the validated Arabic version of the Depression Anxiety Stress Scale (DASS-21) (Moussa et al., 2017). The scale comprises three subscales reflecting depressive, anxious, and stress-related symptoms, respectively. Each subscale consists of 7 items rated on a scale ranging from 0 (did not apply to me at all) to 3 (applied to me most of the time). Subscale scores were summed and then multiplied by 2 to render them comparable with those of the original DASS-42. Severity was categorized as normal, mild, moderate, severe, or extremely severe for each domain. The internal reliability of the DASS-21 in the present study was satisfactory (Cronbach’s α was 0.96 for the total scale, 0.87 for depression, 0.88 for anxiety, and 0.88 for stress). Scores were subsequently classified into two clinically meaningful groups: a low-risk group (normal to mild symptoms) and a high-risk group (moderate to extremely severe symptoms). This dichotomization enabled clinically applicable risk prediction.

This approach was adopted to distinguish participants with at least a moderate symptom burden, a threshold commonly regarded as more clinically meaningful in DASS-21-based analyses, while also supporting stable regression modeling across categories. Accordingly, the binary outcomes were defined using the standard DASS-21 severity thresholds as follows: depression, normal to mild (0–13) versus moderate to extremely severe (≥14); anxiety, normal to mild (0–9) versus moderate to extremely severe (≥10); and stress, normal to mild (0–18) versus moderate to extremely severe (≥19) (Lovibond & Lovibond, 2011).

Predictor Variables: The predictor variables included the following:

- Socio-demographic characteristics (age, gender, parents’ education and occupation, family income, number of family members, and smoking status).
- Gaming addiction was assessed using the 21-item Gaming Addiction Scale (GAS) developed by Lemmens et al. (2009) and translated into Arabic and validated by Asaad et al. (2019). The instrument comprises 7 domains, each containing three items scored on a 5-point scale (never = 1, almost never = 2, sometimes = 3, often = 4, and very often = 5), giving a total score ranging from 21 to 105. The items cover seven addiction criteria (salience, tolerance, mood modification, relapse, withdrawal, conflict, and problems). Adolescents were classified as addicted gamers (all 7 criteria endorsed), excessive gamers (at least 4 of the 7 criteria endorsed), or non-problem gamers (fewer than 4 criteria endorsed) (Asaad et al., 2019; Lemmens et al., 2009). The scale demonstrated acceptable internal consistency in the present study, with a Cronbach’s alpha of 0.811.
- Gaming-related characteristics, including the number of days played per week, gaming hours per day, gaming device, online multiplayer gaming, place of gaming, parental supervision during play, imitation of games in real life, and game genre.

Violent video game playing was operationally defined on the basis of the content of the participant’s most frequently played video game. Participants were asked to report the name of the game they played most often, and the reported games were subsequently classified by the researchers as violent or non-violent. A game was considered violent if its content involved intentional physical harm to other characters, including physical combat, shooting, weapon use, killing, or other actions intended to injure or harm another character. Classification was based on the violence-related content of the reported game and was guided by recognized video game content descriptors (AlHazzaa et al., 2023).

### 2.4. Statistical Analysis

Descriptive statistics were computed as frequencies and percentages, and a significance level of 5% was applied to all analyses. Continuous variables were tested for normality using the Shapiro–Wilk test, and all analyses were performed using SPSS (version 24; IBM Corporation, Armonk, NY, USA). The chi-square test was used to examine the association between categorical variables and the binary outcomes of depression, anxiety, and stress. Fisher’s exact test was applied when expected cell counts were less than 5 in more than 20% of cells. Scores on the Depression, Anxiety, and Stress Scale (DASS-21) were dichotomized, with normal-to-mild symptoms coded as 0 and moderate-to-extremely severe symptoms coded as 1. Univariate analyses were conducted to identify the participant and gaming characteristics significantly associated with each outcome. Candidate variables were selected a priori on the basis of the literature and their theoretical relevance and were subsequently entered into multivariable binary logistic regression models for each outcome (depression, anxiety, and stress).

All pre-specified candidate predictors based on prior literature, theoretical considerations, and their plausible relevance to adolescent mental health and video-gaming behavior, rather than solely on statistical significance, were entered simultaneously into the multivariable binary logistic regression models using the Enter method. This approach was adopted to estimate the independent association of each predictor with the outcome while adjusting for the other variables included in the model. Thus, the adjusted effect estimate for each confounder was obtained while accounting for the other variables included in the model. No preliminary univariable significance threshold or automated stepwise procedure was used to determine inclusion in the multivariable models.

The predictors were coded as follows: age (<17 years = 1, ≥17 years = 0); gender (female = 1, male = 0); violent video game playing (yes = 1, no = 0); gaming hours (>4 h/day = 1, ≤4 h/day = 0); gaming days (daily = 1, non-daily = 0); online multiplayer gaming (yes = 1, no = 0); usual place of gaming (home = 1, outside home = 0); gaming device (portable = 1, non-portable = 0); imitation of games in real life (yes = 1, no = 0); and parental supervision or involvement during gaming (absent = 1, present = 0). The reference category for each binary predictor was coded as 0, and the video game addiction score was entered into the models as a continuous variable.

No subgroup analyses or interaction terms were examined, as these analyses were not pre-specified and the models were developed primarily to estimate the main effects of the candidate predictors.

The Hosmer–Lemeshow goodness-of-fit test was used to assess model assumptions and fit, and all models showed acceptable fit. Linearity in the logit was also tested. Collinearity statistics (VIF < 10) indicated that multicollinearity was not a concern, and the analytical dataset contained neither missing data nor outliers.

Logistic regression was subsequently used to derive the risk scores. Each score was constructed following the method of Sullivan et al. (2004), whereby the β coefficients of the regression model were used to assign points to each significant predictor by dividing its β coefficient by the smallest β coefficient in the model and rounding to the nearest integer. The at-risk category received the assigned points, while its reference category received 0 points. A total numeric risk score for depression, anxiety, and stress was then computed for each individual by summing the points across the variables. Statistical significance was set at *p* < 0.05 for all analyses.

Internal validation of the scores was performed using a bootstrap procedure in which 1000 samples were drawn from the original sample with replacement, the complete model-fitting procedure was repeated within each of the 1000 bootstrap samples, and the performance of the bootstrap models was assessed (Harrell, 2015). In addition, the discriminatory accuracy of each score was evaluated by plotting the ROC curve and computing the corresponding AUC, while calibration was assessed using the Hosmer–Lemeshow test and the calibration plot. The prediction models are reported in accordance with the TRIPOD reporting guidelines (Supplementary Materials).

## 3. Results

A total of 500 eligible adolescent video-game players were invited to participate. Of these, 34 did not complete the questionnaire or declined to participate. Therefore, 466 participants were included in the final analysis (Figure 1). Among the included participants, 41.2% were aged 12–14 years and 47.6% were aged 15–16 years. The majority (62.0%) were male, and 73.8% were enrolled at the intermediate level. Regarding paternal education, 48.5% of fathers had completed secondary education and 42.1% held a university degree or higher, whereas 33.9% of mothers had completed secondary education and 43.8% held a university degree or higher. The largest proportion of participants (42.5%) came from families with a medium monthly income of 10,001–20,000 Saudi Riyals, and approximately 96% of adolescents reported no tobacco use. More than half (51.9%) belonged to medium-sized families of 6–7 members, while 33.5% belonged to large families of 8 or more members (Table 1).

### 3.1. Prevalence of Gaming Addiction, Depression, Anxiety, and Stress

Approximately 43% (*n* = 191) of participants were classified as addicted gamers, whereas 24.5% (*n* = 109) were classified as excessive gamers and 32.6% (*n* = 146) as non-problem gamers (Figure 2).

The prevalence of moderate-to-extremely severe symptoms was 29.2% (*n* = 136) for depression, 47.6% (*n* = 222) for anxiety, and 27.5% (*n* = 128) for stress (Figure 3).

### 3.2. Assessment of Risk Factors for Depression, Anxiety, and Stress Symptoms

Sociodemographic factors: Table 2 showed a significant association between age and depression symptoms (*p* = 0.001); adolescents younger than 17 years had a higher proportion of moderate-to-extremely severe depression symptoms. Females also showed markedly higher rates of moderate-to-extremely severe depression symptoms (*p* < 0.001). Moreover, adolescents at the intermediate educational level showed a significant association with moderate-to-extremely severe depression symptoms (*p* < 0.001). Parental education, family income, number of family members, and smoking were not significantly associated with depression symptoms.

With respect to anxiety, only gender was significantly associated with the level of anxiety symptoms (*p* < 0.001), with females showing a higher proportion of moderate-to-extremely severe anxiety symptoms than males. A significant association was also observed between age and stress level (*p* < 0.001); adolescents younger than 17 years had a higher proportion of moderate-to-extremely severe stress symptoms. Gender was likewise significantly associated with stress symptoms (*p* < 0.001), with females showing a higher proportion of symptoms, and educational level was significantly associated with stress symptoms (*p* = 0.003), with adolescents in the intermediate grades showing a higher proportion of moderate-to-extremely severe stress symptoms. Maternal education and family income were both significantly associated with stress symptoms among adolescents (*p* = 0.044 and *p* = 0.004, respectively), whereas paternal education, number of family members, and smoking showed no significant association with the level of stress symptoms.

Gaming-related characteristics: Table 3 showed a significant association between moderate-to-extremely severe depression symptoms and gaming hours, gaming at home, and playing violent video games (*p* < 0.05), as well as a significant but weak positive correlation between depression symptoms and the gaming addiction score (*p* = 0.048). Moderate-to-extremely severe anxiety symptoms were significantly associated with gaming hours per day (*p* < 0.001), parental supervision during play (*p* = 0.007), gaming at home (*p* = 0.002), use of a portable device (*p* = 0.004), and playing violent video games (*p* < 0.001). There was a significant weak positive correlation between anxiety symptoms and the gaming addiction score (*p* = 0.019). With respect to stress, moderate-to-extremely severe stress symptoms were significantly associated with gaming hours per day (*p* = 0.030), gaming at home (*p* = 0.034), use of a portable device (*p* = 0.006), and playing violent video games (*p* = 0.012), and a significant weak positive correlation was found between stress symptoms and the gaming addiction score (*p* = 0.016).

The logistic regression models (Table 4): Significant predictors of moderate-to-extremely severe depressive symptoms included female gender, gaming at home, playing violent games, and gaming for more than 4 h per day. Females had 4.7 times higher odds of moderate-to-extremely severe depressive symptoms than males, and adolescents who played at home had 1.9 times higher odds than those who played in a café or elsewhere. Those who played violent games had 3.1 times higher odds of moderate-to-extremely severe depressive symptoms than those who did not, and adolescents who played video games for more than 4 h per day had 1.6 times higher odds than those who played for shorter durations.

Significant predictors of moderate-to-extremely severe anxiety symptoms included female gender, gaming for more than 4 h per day, use of portable gaming devices, playing violent games, and age younger than 17 years. Females had 3.7 times higher odds of moderate-to-extremely severe anxiety symptoms than males, and participants who played video games for more than 4 h per day had 2.3 times higher odds than those who played for shorter durations. Portable gaming device users had 1.6 times higher odds of anxiety symptoms than non-users, and participants who played violent games had 2.9 times higher odds than those who did not. In addition, adolescents younger than 17 years had 1.5 times higher odds of moderate-to-extremely severe anxiety symptoms than those aged 17 years or older.

Significant predictors of moderate-to-extremely severe stress symptoms included female gender, age younger than 17 years, gaming for more than 4 h per day, use of portable gaming devices, playing violent games, and online multiplayer gaming. Females had 7.8 times higher odds of moderate-to-extremely severe stress symptoms than males, and adolescents younger than 17 years had 1.6 times higher odds than those aged 17 years or older. Participants who played video games for more than 4 h per day had 4.3 times higher odds of stress symptoms than those who played for shorter durations. Portable gaming device users had 1.5 times higher odds, and participants who played violent games had 2.3 times higher odds of stress symptoms than their respective reference groups, while online multiplayer gaming was associated with 1.9 times higher odds of moderate-to-extremely severe stress symptoms.

Internal validation of score: The assigned scores for depression, anxiety, and stress symptoms were internally validated using receiver operating characteristic (ROC) curve analysis. The AUC for the depression score was 0.780 (95% CI 0.734–0.825), with a sensitivity of 80.1% and a specificity of 62.4% at the optimal cutoff value of 4 (a score ≥ 4 indicating moderate-to-extremely severe depressive symptoms). The AUC for anxiety was 0.799 (95% CI 0.759–0.839), with a sensitivity of 79.3% and a specificity of 68.0% at the optimal cutoff value of 5 (a score ≥ 5 indicating moderate-to-extremely severe anxiety symptoms). The AUC for stress was 0.821 (95% CI 0.778–0.864), with a sensitivity of 83.6% and a specificity of 70.1% at the optimal cutoff value of 7 (a score ≥ 7 indicating moderate-to-extremely severe stress symptoms). The risk score ranged from 0 to 7 for depression, from 0 to 10 for anxiety, and from 0 to 13 for stress symptoms. The three scoring systems demonstrated acceptable-to-good discriminative power, as shown in Figure 4.

The calibration plots (Figure 5): The calibration curve for depression symptoms indicated acceptable calibration with mild overfitting (optimism-corrected slope = 0.866 and intercept = −0.091). For anxiety symptoms, the calibration curve indicated generally good calibration of the anxiety model, with an optimism-corrected calibration slope of 0.887 and an intercept of −0.010. For stress, the optimism-corrected calibration slope was 0.884, and the intercept was −0.080, again suggesting mild overfitting. The Hosmer–Lemeshow goodness-of-fit test was non-significant for all three models (*p* = 0.274, 0.376, and 0.053, respectively), indicating adequate calibration.

Bootstrap internal validation (Table 5) showed that the three models retained acceptable discriminatory performance after correction for optimism. The apparent AUCs were 0.780, 0.799, and 0.821 for the depression, anxiety, and stress models, respectively, with corresponding optimism-corrected AUCs of 0.754, 0.776, and 0.799. The optimism-corrected calibration slopes were 0.866, 0.887, and 0.884, respectively, suggesting mild overfitting, and the calibration intercepts were −0.091, −0.010, and −0.080. Overall, the internal validation results indicate that the models achieved acceptable discrimination and generally satisfactory calibration after adjustment for optimism.

Regression equations for all factors included in the model:

$$P(outcome)=\frac{1}{1+{exp}^{-(Linear\;equation)}}$$

$$\begin{array}{cc} Linear\;equation & for\;depression\\ & =-3.725+1.568\;gender+0.660\;gaming\;place+0.500\;gaming\;hours\\ & +\;1.146\;violent\;game-0.316\;device\;type+0.118\;online\;multiplayer\\ & -\;0.323\;parent\;supervision+0.138\;age+0.045\;days+0.352\;imitate\;game\\ & +\;0.014\;game\;addiction\;score \end{array}$$

$$\begin{array}{cc} Linear\;equation & for\;anxiety\\ & =-1.397+1.314\;gender+0.286\;gaming\;place+0.833\;gaming\;hours\\ & +\;1.049\;violent\;game-0.456\;device\;type-0.424\;online\;multiplayer\\ & -\;0.932\;parent\;supervision+0.405\;age+0.241\;days+0.221\;imitate\;game\\ & +\;0.023\;game\;addiction\;score \end{array}$$

$$\begin{array}{cc} Linear\;equation & for\;stress\\ & =-5.413+2.062\;gender+0.282\;gaming\;place+1.418\;gaming\;hours\\ & +\;0.822\;violent\;game+0.455\;device\;type+0.645\;online\;multiplayer\\ & -\;0.242\;parent\;supervision+0.494\;age+0.431\;days+0.145\;imitate\;game\\ & +\;0.027\;game\;addiction\;score \end{array}$$

## 4. Discussion

In the present study, the prevalence of video gaming addiction among Saudi adolescent video game players was 43%, which is comparable to the 43.9% reported in a Saudi study by Abolfotouh and Barnawi (2024) that used the same instrument to assess gaming addiction. However, this prevalence was lower than that reported in two other Saudi studies (approximately 60%) (Alfaifi et al., 2022; Alrahili et al., 2023). These differences may be explained by the use of different tools to assess gaming addiction and by variations in sample characteristics such as age and gender, as well as by the timing of data collection, whether during vacation or the academic year. The prevalence observed in the present study is also higher than the global pooled prevalence of VG addiction reported in a recent systematic review (8.9%) (Gao et al., 2022). VG addiction therefore appears to constitute a significant problem in Saudi Arabia and raises concern regarding its adverse impact on adolescent health.

The present study revealed a significant positive correlation between gaming addiction and depression, anxiety, and stress symptoms. However, the strength of these correlations was weak, indicating that gaming addiction may account for only a limited proportion of the variance in these mental health outcomes. This finding is consistent with two systematic reviews reporting associations between problematic or addictive video gaming and adverse mental health outcomes, particularly anxiety and depression (Limone et al., 2023; Suratmini, 2024). These associations have been reported across different populations and study designs. A probable explanation for this relationship is that excessive time spent gaming may lead to social isolation, which is in turn associated with impaired psychological well-being and greater susceptibility to emotional distress.

Despite this association, adolescents differ in their degree of vulnerability according to sociodemographic and gaming characteristics. Therefore, the present study aimed to develop and internally validate multivariable models for identifying adolescent video game players with a higher probability of concurrent moderate-to-extremely severe depression, anxiety, and stress symptoms. To the best of our knowledge, no such risk score is currently available, and its development may enhance timely screening, targeted intervention, and the efficient allocation of mental health resources.

The present study developed and internally validated multivariable models for the concurrent identification of moderate-to-extremely severe depression, anxiety, and stress symptoms. Overall, the models showed acceptable-to-good discrimination, with apparent AUCs of 0.780 for depression, 0.799 for anxiety, and 0.821 for stress; the stress model thus demonstrated the strongest discriminatory performance, followed by the anxiety and depression models. Internal validation using 1000 bootstrap resamples also indicated generally satisfactory calibration, with optimism-corrected calibration slopes of 0.866 for depression, 0.887 for anxiety, and 0.884 for stress and corresponding calibration intercepts of −0.091, −0.010, and −0.080, indicating satisfactory calibration with only mild overfitting.

The relatively better performance of the stress model may be attributable to the fact that depression and anxiety are multifactorial psychological outcomes influenced by a broader range of determinants. Consequently, the ability of a relatively simple model based largely on routinely obtainable gaming and demographic characteristics to achieve AUCs of approximately 0.78–0.82 is potentially meaningful for preliminary screening, although the models should not be regarded as diagnostic tools. Comparison of the present models with other published prediction models among gamers is difficult, because most previous studies have focused on predicting gaming addiction itself rather than depression, anxiety, or stress as outcomes.

A study by Aggarwal et al. (2020) developed a model to predict generalized anxiety disorder that achieved a classification accuracy of 84.9%. Although this reported accuracy is higher than the performance of the present anxiety model, the two values are not directly comparable with an AUC of 0.799, because classification accuracy and AUC are fundamentally different measures. Moreover, Aggarwal et al. used detailed gaming characteristics and machine-learning algorithms, whereas the present study relied on readily obtainable variables applicable across different types of video games.

For anxiety, the study by Hein et al. (2024) examined whether gamer–avatar relationships, age, and gaming duration could be combined with artificial intelligence classifiers to assess both concurrent and prospective anxiety among gamers. The present anxiety model differs from that approach in both its intended use and its predictor variables; nevertheless, it achieved satisfactory performance while offering a simpler and more interpretable screening approach.

For depression, a study conducted by Lundeen and McCall (2025) studied 340 online gamers aged 18–24 years. Their study used behavioral, psychosocial, and gaming characteristics to predict major depressive disorder, whereas the present depression model targeted adolescents and relied on demographic and gaming characteristics.

An important feature of the three scores developed in the present study is the inclusion of both non-modifiable and modifiable predictors. Age and gender are demographic characteristics that may identify groups with differing baseline susceptibility but are not themselves intervention targets. In contrast, gaming duration in hours, violent-game exposure, online multiplayer gaming, use of portable gaming devices, and place of gaming represent modifiable predictors. Although the depression, anxiety, and stress models share several risk factors, these factors do not carry the same relative importance or contribute to the same degree across the three outcomes. Separate outcome-specific scores are therefore more appropriate than a single combined mental health score.

The present study also revealed that female gender was a common and consistent component of the concurrent risk scores for depression, anxiety, and stress symptoms, indicating that female adolescents are more vulnerable to gaming-related psychological problems. This finding aligns with previous evidence and may be explained by biological factors, including hormonal fluctuations during puberty, together with psychological and social influences such as greater emotional sensitivity and stress reactivity (Liao et al., 2025).

In the present study, violent video game playing was a common predictor across the depression, anxiety, and stress models. A previous systematic review reported a relationship between violent gaming and aggressive behavior but did not examine its association with depression, anxiety, or stress symptoms (AlHazzaa et al., 2023). The present findings therefore add to the literature by showing that violent-game exposure contributes to multivariable risk classification for all three psychological outcomes. Nevertheless, the models support its role as a shared marker for concurrent risk stratification rather than establishing it as a causal determinant.

Age younger than 17 years was associated with an increased concurrent likelihood of moderate-to-extremely severe anxiety and stress symptoms but was not a significant predictor of depression, suggesting that younger adolescent gamers may be particularly vulnerable to anxiety- and stress-related symptoms.

The three derived risk scores demonstrated acceptable-to-good discriminative ability for identifying moderate-to-extremely severe depression, anxiety, and stress symptoms among adolescent video game players. The stress score showed the strongest discrimination (AUC = 0.821, 95% CI 0.778–0.864), followed by the anxiety score (AUC = 0.799, 95% CI 0.759–0.839) and the depression score (AUC = 0.780, 95% CI 0.734–0.825). At a cutoff of ≥7, the stress score achieved the most favorable classification performance, with a sensitivity of 83.6% and a specificity of 70.1%. At a cutoff of ≥5, the anxiety score achieved a sensitivity of 79.3% and a specificity of 68.0%, whereas the depression score, at a cutoff of ≥4, achieved a sensitivity of 80.1% but a comparatively lower specificity of 62.4%. The relatively high sensitivity and moderate specificity, particularly for depression symptoms, indicate that positive classifications should prompt further assessment rather than being interpreted as diagnostic. External validation is therefore required before these cutoff values can be recommended for school-based screening.

The developed scores are of practical value because they rely on readily obtainable demographic and gaming-related characteristics, which makes them potentially feasible for use in schools as screening rather than diagnostic or prognostic instruments. Adolescents exceeding the selected thresholds of ≥4 for depression, ≥5 for anxiety, and ≥7 for stress could be prioritized for further professional assessment and clinical follow-up.

Limitations and Future Research: The present study should be interpreted in light of several limitations. The cross-sectional design limits the ability to establish causality; although associations can be identified, it is not possible to determine the directionality of the relationships between depression, anxiety, and stress symptoms and their predictors. Recall bias was minimized but could not be eliminated entirely; to reduce it, a short and clearly specified recall period with simple, predefined response categories was used. Because the predictors and outcomes were assessed simultaneously using self-reported data, common-method bias may also have been introduced. Several procedural measures were implemented to minimize this bias, including the use of validated instruments to assess both predictors and outcomes, the presentation of predictor and outcome measures in separate sections of the questionnaire, and the assessment of gaming-related characteristics using clearly defined items and simple wording. Finally, conducting the study in a single region (Aseer) may limit the generalizability of the findings to adolescents across Saudi Arabia, warranting external validation in other regions.

Subgroup and interaction analyses were not performed because they were not pre-specified in the model. Future studies should assess clinically relevant interactions and subgroup analysis. In addition, future research should focus on the external validation of the developed screening models in independent and more diverse adolescent populations in order to assess their generalizability and clinical utility. Also, the precision of estimated scores can be obtained through external validation.

## 5. Conclusions

Overall, the three internally validated models demonstrated acceptable-to-good discrimination and generally satisfactory calibration, with the stress model performing best in terms of discrimination. In addition, the present study translated the multivariable analysis logistic regression models into practical scoring tools. These tools may support the identification of adolescent video game players at current risk of moderate-to-extremely severe depression, anxiety, and stress symptoms, thereby facilitating timely identification and appropriate intervention for adolescents at risk of poorer mental health outcomes. External validation, prospective evaluation, and assessment of clinical utility are required before routine implantation in schools, clinics, or digital platforms.

## Figures and Tables

**Figure 1:**
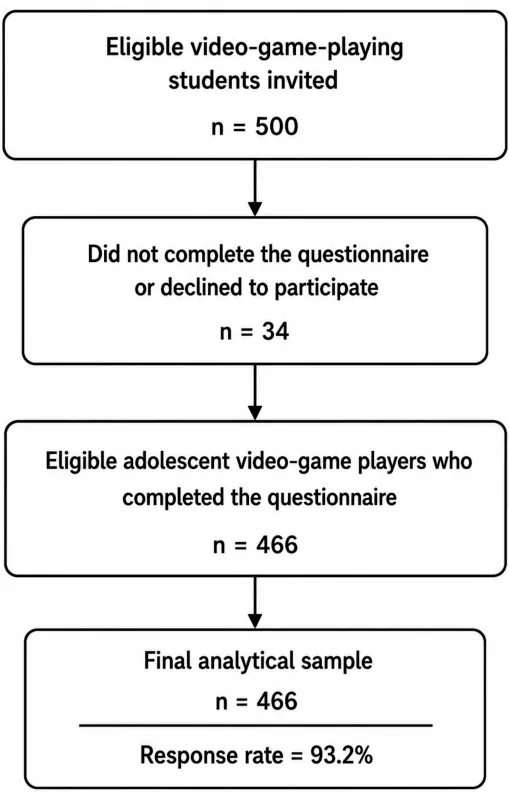
Participant flow and exclusion diagram.

**Figure 2:**
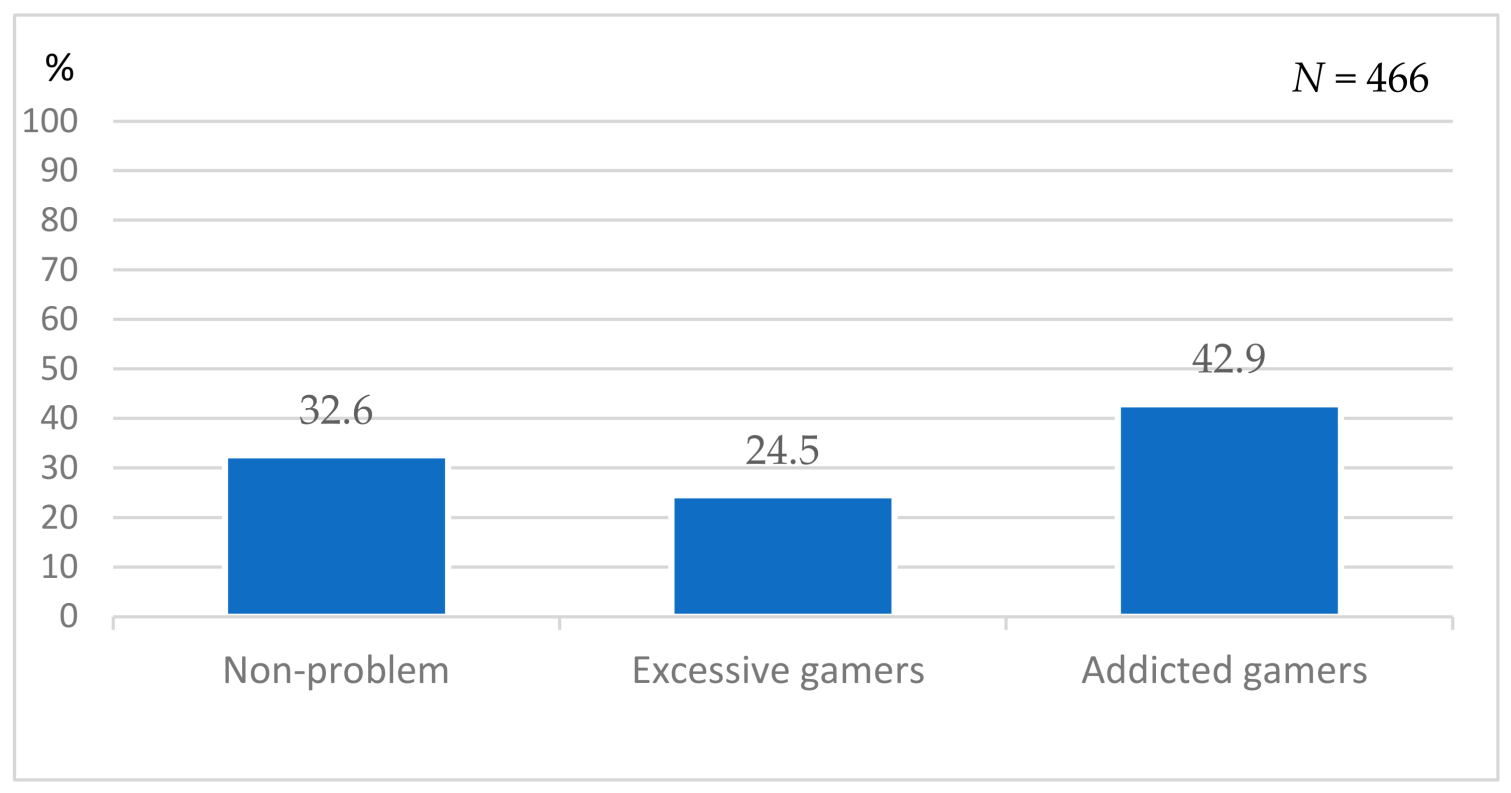
Levels of gaming addiction among Saudi adolescent video game players.

**Figure 3:**
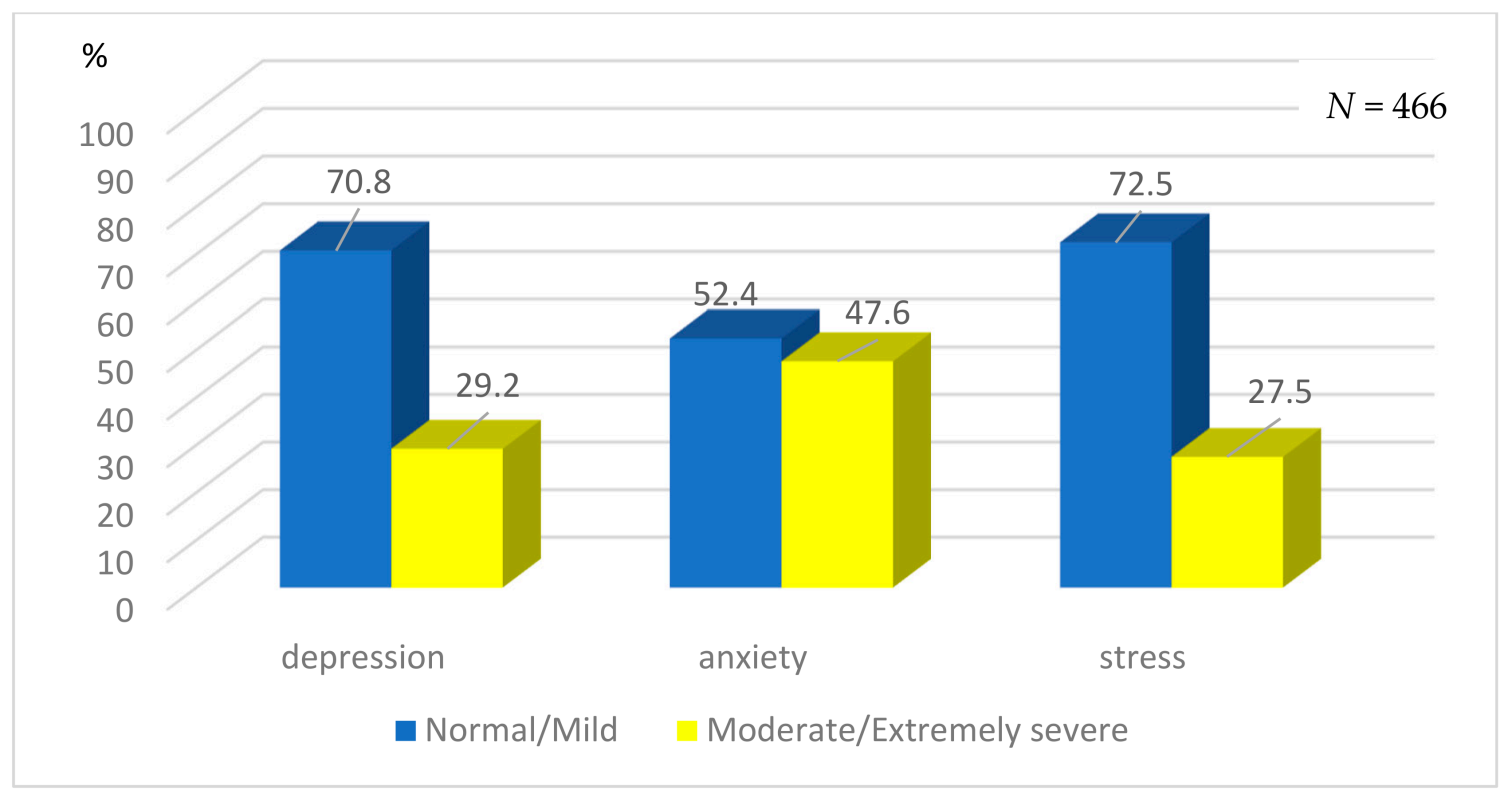
Levels of depression, anxiety, and stress among Saudi adolescent video game players.

**Figure 4:**
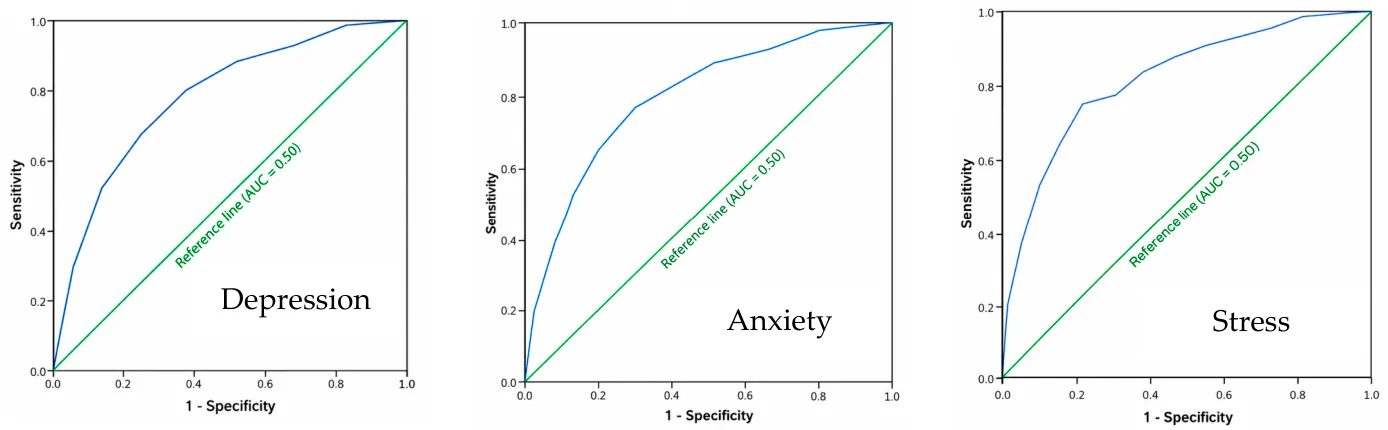
ROC curve for the three scoring schemes.

**Figure 5:**
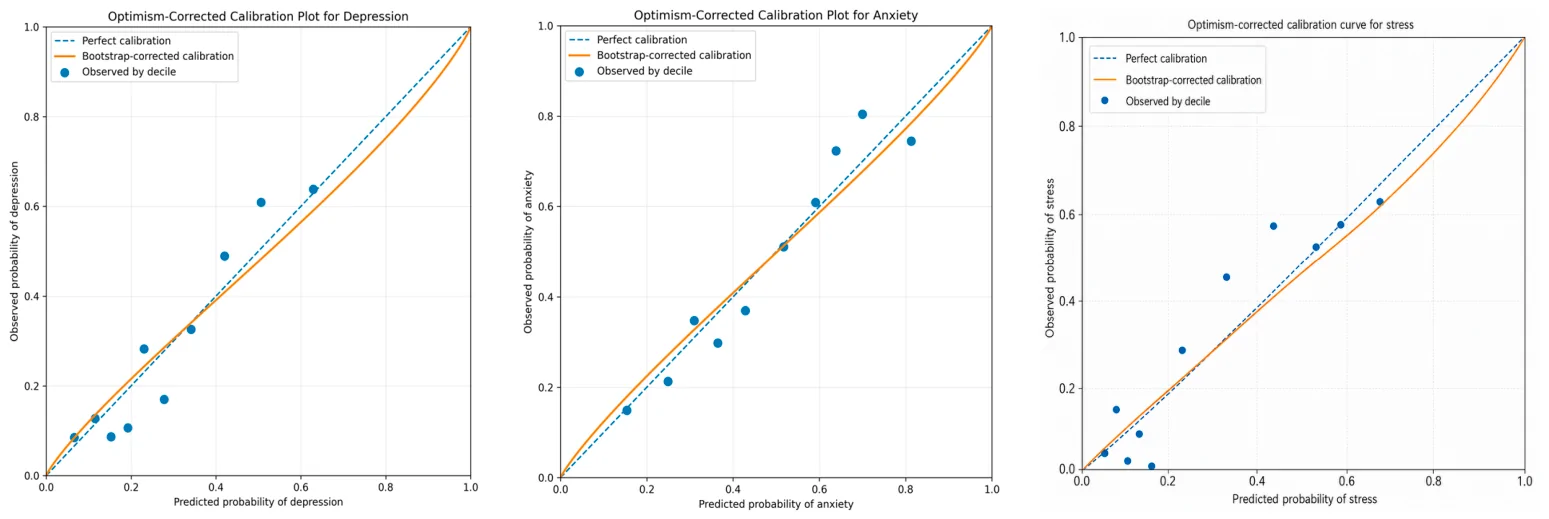
Calibration plot for the three scoring schemes.

**Table 1:** Socio-demographic characteristics of the study participants.

Characteristics	N (%) N = 466
**Age**	
12–14 years	192 (41.2%
15–16 years	222 (47.6%)
17–18 years	52 (11.2%)
**Gender**	
Female	177 (38.0%)
Male	289 (62.0%)
**Level**	
Intermediate	344 (73.8%)
Secondary	122 (26.2%)
**Father’s Education**	
Below Secondary	44 (9.4%)
Secondary	226 (48.5%)
University or above	196 (42.1%)
**Mather’s Education**	
Below Secondary	104 (22.3%)
Secondary	158 (33.9%)
University or above	204 (43.8%)
**Family Income**	
<10,000 SR (low)	126 (27.0%)
10,000–20,000 SR (medium)	198 (42.5%)
20,000 or more SR (high)	142 (30.5%)
**Tobacco use**	
No	448 (96.1%)
Yes	18 (3.9%)
**Number of Family Members**	
4–5	68 (14.6%)
6–7	242 (51.9%)
8+	156 (33.5%)

**Table 2:** Association of sociodemographic characteristics with depression, anxiety, and stress among Saudi adolescent video game players (*n* = 466).

Characteristics	Depression			Anxiety			Stress		
	Normal/Mild (*n* = 330)	Moderate/ Extremely Severe (*n* = 136)	*p*-Value	Normal/Mild (*n* = 244)	Moderate/ Extremely Severe (*n* = 222)	*p*-Value	Normal/Mild (*n* = 338)	Moderate/Extremely Severe (*n* = 128)	*p*-Value
**Age**									
12–14 years	146 (44.2%)	46 (33.9%)	0.001 *	104 (42.6%)	88 (39.6%)	0.299	158 (46.7%)	34 (26.6%)	<0.001 *
15–16 years	158 (47.9%)	64 (47.1%)		118 (48.4%)	104 (46.8%)		152 (45.0%)	70 (54.7%)	
17–18 years	26 (7.9%)	26 (19.0%)		22 (9.0%)	30 (13.6%)		28 (8.3%)	24 (18.8%)	
**Gender**									
Female	87 (26.4%)	90 (66.2%)	<0.001 *	56 (23.0%)	121 (54.5%)	<0.001 *	84 (24.9%)	93 (72.7%)	<0.001 *
Male	243 (73.6%)	46 (33.8%)		188 (77.0%)	101 (45.5%)		254 (75.1%)	35 (27.3%)	
**Level of education**									
Intermediate	260 (78.7%)	84 (61.8%)	<0.001 *	194 (79.5%)	150 (67.5%)	0.989	262 (77.5%)	82 (64.1%)	0.003 *
Secondary	70 (21.2%)	52 (38.2%)		50 (20.5%)	72 (32.5%)		76 (22.5%)	46 (35.9%)	
**Father’s Education**									
Below Secondary	40 (9.1%)	14 (10.3%)		22 (9.0%)	22 (9.9%)	0.209	36 (10.6%)	8 (6.2%)	0.325
Secondary	160 (48.5%)	66 (48.5%)	0.912	110 (45.1%)	116 (52.2%)		160 (47.3%)	66 (51.6%)	
University or above	140 (42.4%)	56 (41.2%)		112 (45.9%)	84 (37.9%)		142 (42.0%)	54 (43.2%)	
**Mather’s Education**									
Below Secondary	82 (24.8%)	22 (16.2%)	0.124	60 (24.5%)	44 (19.8%)	0.465	80 (23.7%)	24 (18.7%)	0.044 *
Secondary	108 (33.9%)	50 (36.8%)		80 (32.7%)	78 (35.2%)		122 (36.1%)	36 (28.1%)	
University or above	140 (43.8%)	64 (47.1%)		104 (42.6%)	100 (45.0%)		136 (40.2%)	68 (53.1%)	
**Family Income**									
<10,000 SR (low)	90 (27.3%)	36 (26.5%)	0.060	76 (31.1%)	50 (22.5%)	0.861	96 (28.4%)	30 (34.4%)	0.004 *
10,000–20,000 SR (Medium)	130 (39.4%)	68 (50.0%)		80 (32.7%)	118 (53.2%)		128 (37.9%)	70 (54.7%)	
20,000+ SR (high)	110 (33.3%)	32 (23.5%		88 (36.2%)	54 (28.3%)		114 (33.7%)	28 (21.9%)	
**Tobacco use**									
No	318 (96.4%)	130 (95.6%)	0.709	236 (96.7%)	212 (95.5%)	0.493	326 (96.4%)	122 (95.3%)	0.570
Yes	12 (3.6%)	6 (4.4%)		8 (3.3%)	10 (4.5%)		12 (3.6%)	6 (4.7%)	
**Number of Family Members**									
4–5	52 (15.7%)	16 (11.8%)	0.526	34 (13.9%)	34 (15.3%)	0.861	52 (15.4%)	16 (12.5%)	0.653
6–7	168 (50.9%)	74 (54.4%)		126 (51.6%)	116 (52.3%)		176 (52.1%)	66 (51.6%)	
**8+**	110 (33.4%)	46 (33.8%)		84 (34.5%)	72 (32.4%)		110 (32.5%)	46 (35.9%)	

*χ*^2^ Test: Chi-square test for association. Exact test: used when expected frequencies are less than 5. * Significance: (*p* < 0.05).

**Table 3:** Associations of gaming characteristics with depression, anxiety, and stress among Saudi adolescent video game players (*n* = 466).

Characteristics	Depression			Anxiety			Stress		
	Normal/Mild (*n* = 330)	Moderate/Extremely Severe (*n* = 136)	*p*-Value	Normal/Mild (*n* = 244)	Moderate/ Extremely Severe (*n* = 222)	*p*-Value	Normal/Mild (*n* = 338)	Moderate/Extremely Severe (*n* = 128)	*p*-Value
**Gaming days**									
<2 days	115 (34.8%)	61 (44.9%)	0.079	90 (36.9%)	86 (38.7%)	0.148	123 (36.4%)	53 (41.4%)	0.135
3–4 days	107 (32.4%)	32 (23.5%)		81 (33.2%)	58 (26.1%)		111 (32.8%)	28 (21.9%)	
5–6 days	34 (10.3%)	9 (6.6%)		25 (10.2%)	18 (8.1%)		31 (9.2%)	12 (9.4%)	
Daily	74 (22.4%)	34 (25.0%)		48 (19.7%)	60 (27.0%)		73 (21.6%)	35 (27.3%)	
**Gaming hours**									
<1 h	40 (12.1%)	17 (12.5%)	0.048 *	29 (11.9%)	28 (12.6%)	<0.001 *	34 (10.1%)	23 (18.0%)	0.030 *
1–2 h	137 (41.5%)	47 (34.6%)		124 (50.8%)	60 (27.0%)		145 (42.9%)	39 (30.5%)	
3–4 h	81 (24.5%)	26 (19.1%)		48 (19.7%)	59 (26.6%)		77 (22.8%)	30 (23.4%)	
>4 h	72 (21.8%)	46 (33.8%)		43 (17.6%)	75 (33.8%)		82 (24.3%)	36 (28.1%)	
**Online multiplayer**									
No	77 (23.3%)	23 (16.9%)	0.142	54 (22.1%)	46 (20.7%)	0.711	72 (21.3%)	28 (21.9%)	0.893
Yes	253 (76.7%)	113 (83.1%)		190 (77.9%)	176 (79.3%)		266 (78.7%)	100 (78.1%)	
**Parent supervision during play**									
No	52 (15.8%)	25 (18.4%)	0.488	29 (11.9%)	48 (21.6%)	0.007 *	54 (16.0%)	23 (18.0%)	0.605
Yes	278 (84.2%)	111 (81.6%)		215 (88.1%)	174 (78.4%)		284 (84.0%)	105 (82.0%)	
**Gaming place**									
Home	127 (38.5%)	81 (59.6%)	<0.001 *	92 (37.7%)	116 (52.3%)	0.002 *	140 (41.4%)	68 (53.1%)	0.034 *
Café/Friend’s/Other	203 (61.5%)	55 (40.4%)		152 (62.3%)	106 (47.7%)		198 (58.6%)	60 (46.9%)	
**Game device**									
Non-portable	107 (32.4%)	39 (28.7%)	0.428	91 (37.3%)	55 (24.8%)	0.004 *	119 (35.2%)	28 (21.9%)	0.006 *
Portable	223 (67.6%)	97 (71.3%)		153 (62.7%)	167 (75.2%)		219 (64.8%)	100 (78.1%)	
**Imitate game in real life**									
No	291 (88.2%)	115 (84.6%)	0.288	215 (88.1%)	191 (86.0%)	0.503	295 (87.3%)	111 (86.7%)	0.872
Yes	39 (11.8%)	21 (15.4%)		29 (11.9%)	31 (14.0%)		43 (12.7%)	17 (13.3%)	
**Game genre**									
Non-violent	74 (22.4%)	11 (8.1%)	<0.001 *	59 (24.2%)	26 (11.7%)	<0.001 *	71 (21.0%)	14 (10.9%)	0.012 *
Violent	256 (77.6%)	125 (91.9%)		185 (75.8%)	196 (88.3%)		267 (79.0%)	114 (89.1%)	
**Gaming Addiction score**	R_s_ coefficient = 0.101		0.047 *	R_s_ coefficient = 0.108		0.019 *	R_s_ coefficient = 0.112		0.016 *

*χ*^2^ Test: Chi-square test for association. Exact test: used when expected frequencies are less than 5. R_s_ (Spearman correlation coefficient). * Significance: (*p* < 0.05).

**Table 4:** Multivariable logistic regression models of the significant factors associated with depression, anxiety, and stress symptoms among Saudi adolescent video game players.

Condition	Variable	β	*p*-Value	AOR	Lower 95% CI	Upper 95% CI	Point Score
**Depression**	Female gender	1.568	<0.001	4.7	3.0	7.7	3
	Gaming at home	0.660	0.006	1.9	1.2	3.1	1
	Gaming more than 4 h/day	0.500	0.049	1.6	1.2	2.7	1
	Playing violent game	1.146	0.006	3.1	1.4	7.1	2
**Anxiety**	Female gender	1.314	<0.001	3.7	2.4	5.8	3
	Gaming more than 4 h/day	0.833	0.001	2.3	1.4	3.7	2
	Portable device users	0.465	0.048	1.6	1.2	2.5	1
	Age less than 17 years	0.405	0.022	1.5	1.3	2.4	1
	Playing violent game	1.049	0.002	2.9	1.5	5.5	3
**Stress**	Female gender	2.062	<0.001	7.8	4.7	9.1	5
	Age less than 17 years	0.494	0.042	1.6	1.2	2.7	1
	Gaming more than 4 h/day	1.418	0.030	4.3	3.2	6.8	3
	Portable device users	0.455	0.013	1.5	1.2	2.8	1
	Playing violent game	0.822	0.043	2.3	1.3	5.1	2
	Online multiplayer	0.645	0.020	1.9	1.004	3.05	1

CI: Confidence Interval. *p*-value: Indicates statistical significance (*p* < 0.05). AOR: Adjusted Odds Ratio.

**Table 5:** Bootstrap analysis of the risk scores for depression, anxiety, and stress symptoms among Saudi adolescent video game players.

	AUC	Estimated AUC Optimism	Optimism—Corrected AUC	Corrected Calibration Slope	Corrected Intercept
**Depression**	0.780	0.026	0.754	0.866	−0.091
**Anxiety**	0.799	0.023	0.776	0.887	−0.010
**Stress**	0.821	0.022	0.799	0.884	−0.080

## Data Availability

Data are available from the corresponding author upon reasonable request.

## Supplementary Materials

The following supporting information can be downloaded at: https://www.mdpi.com/article/10.3390/bs16101731/s1, File S1: TRIPOD Checklist: Prediction Model Development.
